# Supplementary material for: MetaRibo-Seq measures translation in microbiomes
Source: Nat Commun. 2020 Jun 29;11:3268. doi: 10.1038/s41467-020-17081-z (PMC7324362; doi:10.1038/s41467-020-17081-z)
Supplement: Supplementary file 10 — Supplementary Data 7 [file 41467_2020_17081_MOESM10_ESM.zip › File2/Confidence_High_Taxonomy/221334_out.krona.html]

Javascript must be enabled to view this page.

members
magnitude
magnitudeUnassigned
count
unassigned
taxon
rank

221334\_out

173

2
superkingdom
173

976
phylum
173

173
200643
class

171549
order
173

171550
family
2

2
239759
genus


SRS1041134\_contig\_number\_10382SRS105082\_contig\_number\_14932
2
28117
species

12
family
815

12
816
genus


SRS051610\_contig\_number\_9035
1
821
species

28111
species
1

SRS020233\_contig\_number\_41321

1

SRS016753\_contig\_number\_5500
46506
species


SRS023526\_contig\_number\_35261SRS149075\_contig\_number\_5362
2
species
820

1

SRS1041130\_contig\_number\_92
818
species

246787
species
2

SRS045244\_contig\_number\_138SRS045713\_contig\_number\_11672

1263053
species

SRS078242\_contig\_number\_13703
1

1

SRS012969\_contig\_number\_21566
species
329854

species
1638780
2

SRS063190\_contig\_number\_1711SRS148159\_contig\_number\_42458

1
1853231
family

genus
283168
1

species
28118

SRS078176\_contig\_number\_17869
1

2005525
family
157

157
375288
genus

46503
species

SRS011302\_contig\_number\_27957SRS013098\_contig\_number\_7194SRS013521\_contig\_number\_8861SRS013687\_contig\_number\_33955SRS013800\_contig\_number\_23829SRS013940\_contig\_number\_10869SRS013951\_contig\_number\_35072SRS014235\_contig\_number\_32697SRS014287\_contig\_number\_10490SRS014412\_contig\_number\_9981SRS014613\_contig\_number\_11629SRS014683\_contig\_number\_13805SRS014923\_contig\_number\_29220SRS014948\_contig\_number\_20068SRS015264\_contig\_number\_17834SRS015431\_contig\_number\_63120SRS015663\_contig\_number\_24712SRS015694\_contig\_number\_12703SRS015782\_contig\_number\_32440SRS016018\_contig\_number\_contig-100\_1096.96283SRS016056\_contig\_number\_814SRS016132\_contig\_number\_4276SRS016381\_contig\_number\_1468SRS017247\_contig\_number\_2079SRS017859\_contig\_number\_4136SRS018049\_contig\_number\_172SRS018133\_contig\_number\_5966SRS018836\_contig\_number\_13507SRS018936\_contig\_number\_8686SRS019068\_contig\_number\_100087SRS019178\_contig\_number\_4SRS019286\_contig\_number\_1209SRS019381\_contig\_number\_3621SRS019397\_contig\_number\_8630SRS019445\_contig\_number\_20856SRS019685\_contig\_number\_37917SRS019808\_contig\_number\_18477SRS019968\_contig\_number\_8647SRS020328\_contig\_number\_20080SRS022137\_contig\_number\_1044SRS023583\_contig\_number\_2061SRS023914\_contig\_number\_12650SRS024075\_contig\_number\_3202SRS024132\_contig\_number\_45581SRS024435\_contig\_number\_23006SRS024492\_contig\_number\_19926SRS042628\_contig\_number\_37546SRS042966\_contig\_number\_contig-100\_509.131994SRS043841\_contig\_number\_3373SRS044535\_contig\_number\_189SRS045004\_contig\_number\_25589SRS045645\_contig\_number\_3767SRS046068\_contig\_number\_1244SRS046717\_contig\_number\_8614SRS047044\_contig\_number\_17346SRS047433\_contig\_number\_18744SRS048164\_contig\_number\_9043SRS048870\_contig\_number\_8929SRS049773\_contig\_number\_28028SRS049896\_contig\_number\_23050SRS049959\_contig\_number\_37940SRS049995\_contig\_number\_37172SRS050299\_contig\_number\_9560SRS050520\_contig\_number\_23SRS050925\_contig\_number\_2786SRS051882\_contig\_number\_11918SRS052078\_contig\_number\_2795SRS052697\_contig\_number\_44771SRS053356\_contig\_number\_36637SRS053398\_contig\_number\_11312SRS054059\_contig\_number\_4401SRS054352\_contig\_number\_13169SRS055966\_contig\_number\_1178SRS055982\_contig\_number\_97SRS057480\_contig\_number\_2500SRS058070\_contig\_number\_12335SRS058770\_contig\_number\_13529SRS062654\_contig\_number\_6322SRS063127\_contig\_number\_3168SRS064276\_contig\_number\_contig-100\_210.212230SRS064557\_contig\_number\_22975SRS064757\_contig\_number\_3610SRS065176\_contig\_number\_5264SRS065397\_contig\_number\_4873SRS065504\_contig\_number\_30248SRS074670\_contig\_number\_3722SRS075021\_contig\_number\_7836SRS075341\_contig\_number\_18789SRS075773\_contig\_number\_28402SRS075821\_contig\_number\_14046SRS075984\_contig\_number\_11051SRS076876\_contig\_number\_112SRS076976\_contig\_number\_18229SRS077024\_contig\_number\_1506SRS077294\_contig\_number\_12763SRS077552\_contig\_number\_21369SRS077589\_contig\_number\_974SRS077641\_contig\_number\_4953SRS078419\_contig\_number\_28973SRS097958\_contig\_number\_17816SRS098061\_contig\_number\_9543SRS098571\_contig\_number\_51142SRS098644\_contig\_number\_33424SRS098655\_contig\_number\_2419SRS101376\_contig\_number\_27678SRS101433\_contig\_number\_13452SRS103987\_contig\_number\_19027SRS104036\_contig\_number\_6659SRS1041039\_contig\_number\_9151SRS1041092\_contig\_number\_15910SRS1041112\_contig\_number\_2133SRS1041137\_contig\_number\_18594SRS1041138\_contig\_number\_5299SRS1041140\_contig\_number\_9290SRS1041141\_contig\_number\_8058SRS104327\_contig\_number\_6261SRS104636\_contig\_number\_23913SRS104912\_contig\_number\_19952SRS104975\_contig\_number\_4298SRS105153\_contig\_number\_13351SRS1054716\_contig\_number\_5807SRS142503\_contig\_number\_45249SRS143148\_contig\_number\_13765SRS143181\_contig\_number\_11061SRS143372\_contig\_number\_1347SRS143722\_contig\_number\_11146SRS143780\_contig\_number\_24658SRS143991\_contig\_number\_29304SRS144506\_contig\_number\_6211SRS144537\_contig\_number\_40188SRS144714\_contig\_number\_21843SRS145308\_contig\_number\_2250SRS145497\_contig\_number\_14135SRS146764\_contig\_number\_21502SRS146812\_contig\_number\_59852SRS146813\_contig\_number\_19308SRS147139\_contig\_number\_17572SRS147271\_contig\_number\_25299SRS147346\_contig\_number\_24355SRS147425\_contig\_number\_4792SRS147557\_contig\_number\_24651SRS148091\_contig\_number\_9045SRS148196\_contig\_number\_34336SRS148253\_contig\_number\_12365SRS148319\_contig\_number\_4463SRS148721\_contig\_number\_49892SRS148784\_contig\_number\_23323SRS148817\_contig\_number\_contig-100\_213.94111SRS149181\_contig\_number\_23168SRS893230\_contig\_number\_9368SRS893252\_contig\_number\_10253SRS893295\_contig\_number\_6705SRS893295\_contig\_number\_9690SRS893327\_contig\_number\_7404SRS893358\_contig\_number\_12696SRS893378\_contig\_number\_19527
156

1

SRS020508\_contig\_number\_427
1263094
species

1
family
171552

1
genus
577309

species
1263095
1

SRS063985\_contig\_number\_19582
